# Ultrasound-Based Attenuation Imaging for the Non-Invasive Quantification of Liver Fat - A Pilot Study on Feasibility and Inter-Observer Variability

**DOI:** 10.1109/JTEHM.2020.3001488

**Published:** 2020-06-10

**Authors:** Daniel Jesper, Daniel Klett, Barbara Schellhaas, Lukas Pfeifer, Moritz Leppkes, Maximilian Waldner, Markus F. Neurath, Deike Strobel

**Affiliations:** Department of Internal Medicine 1Erlangen University Hospital, FAU University of Erlangen-Nürnberg91054ErlangenGermany

**Keywords:** Attenuation imaging, fatty liver, hepatic fat, hepatic steatosis, hepatorenal index, inter-observer, liver steatosis, NAFLD, NASH, ultrasound

## Abstract

Attenuation imaging is a novel, ultrasound-based technique to objectively detect and quantify liver steatosis. In this study, we evaluated the performance and inter-observer variability of attenuation imaging and compared it to a known quantification method of liver fat, the hepatorenal index (HRI). Two observers measured attenuation coefficients (AC) in an attenuation phantom, 20 healthy volunteers and 27 patients scheduled for biopsy for suspected diffuse liver disease. Results were compared with the HRI and histological findings. Both observers were blinded to the results of the biopsy and the measurements of the other observer. Our results showed that patients with moderate (S2, 33-66%) and severe fatty infiltration of the liver (S3, >66%) showed significantly higher ACs in comparison to patients with a liver fat fraction of less than 33% (S0/1). There was no significant difference in AC-values of patients with fatty infiltration of less than 5% (S0) and 5-32% (S1). In the Receiver Operating Characteristic (ROC)-analysis, the area under the curve (AUC)-values for the detection of moderate and severe steatosis were excellent at 0.98. Cut-off values were 0.64 dB/cm/MHz for the detection of S2- and 0.68 dB/cm/MHz for the detection of S3-steatosis. The inter-observer agreement of attenuation imaging was very good with an intraclass correlation coefficient (ICC) of 0.92 in patient and 0.96 in phantom measurements. The ICC decreased with depth in the phantom measurements. In summary, attenuation imaging showed very good inter-observer agreement and is a promising tool for the detection and quantification of moderate and severe hepatic steatosis.

## Graphical abstract

Attenuation imaging is a novel, ultrasound-based technique to detect and quantify liver steatosis. In this study, we evaluated the performance and inter-observer variability of attenuation imaging and compared it to a known quantification method of liver fat, the hepatorenal index (HRI). Results in 20 healthy volunteers and 27 patients scheduled for biopsy for suspected diffuse liver disease showed very good inter-observer agreement. Based on this study, attenuation imaging is a promising tool for the detection and quantification of moderate and severe hepatic steatosis.

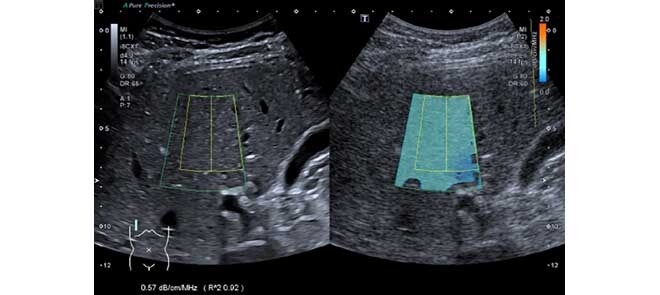

## I. Introduction

The prevalence of non-alcoholic fatty liver disease (NAFLD) has been on the rise during the last decades and has become a large burden for healthcare systems around the world [1]. NAFLD can lead to inflammation, fibrosis and cirrhosis and is a known risk factor for the development of hepatocellular carcinoma (HCC) [2]. Conventional ultrasound (US) is used most commonly to detect the presence of liver steatosis and B-mode image scoring systems can be used to distinguish between different degrees of fatty liver. Although some reports have shown a positive correlation with the severity of hepatic fat accumulation and the presence of complications like non-alcoholic steatohepatitis (NASH) for these scores [3], [4], others have found low inter-observer agreement and accuracy in predicting the correct degree of hepatic steatosis [5]. Therefore, new ultrasound techniques have been developed to objectively quantify the degree of fatty infiltration in the liver. A method which has been used for more than a decade is the computerized measurement of the hepatorenal index (HRI), which compares the brightness of the liver and kidney parenchyma in the B-mode image with the help of computer software. A significantly brighter liver parenchyma indicates fatty infiltration. Studies examining the HRI have yielded different results for the exact cut-off value for the detection of liver steatosis [6], [7]. Controlled Attenuation Parameter (CAP), which is available for FibroScan®(Echosens, Paris, France) measures the attenuation of the ultrasound wave in the liver parenchyma. A correlation with hepatic steatosis has been shown in multiple scientific studies [8]. CAP is not implemented in an ultrasound machine with a B-mode image and requires additional examination with the FibroScan®-system. Since the region of interest (ROI) is not visualized during the measurement, artifacts and areas of heterogeneous liver parenchyma cannot be avoided with this method. A novel technique is attenuation imaging, which has been embedded by Canon Medical Systems (Otawara, Japan) into their current Aplio®-series of ultrasound devices. By measuring the attenuation coefficient (AC) of the ultrasound beam in the liver parenchyma, it relies on the same physical principles as CAP. The AC is supposed to correlate with the degree of steatosis, since a high fraction of intrahepatic fat attenuates the ultrasound signal more than a low amount. To perform a measurement, the AC is color-mapped onto the real time ultrasound B-mode image. The user can then select a region of interest, in which the AC is measured in dB/cm/MHz. Until now, only limited scientific data about the diagnostic performance of attenuation imaging in quantifying fatty infiltration of the liver have been published [9]–[13]. In this prospective pilot study, we examined the inter-observer variability and diagnostic capabilities of attenuation imaging in an ultrasound phantom with known attenuation values, healthy volunteers and patients with suspected diffuse liver disease, who had undergone liver biopsy. We compared the results with a conventional US-scoring system for the quantification of liver fat and the HRI using histopathology as the gold-standard.

## II. Materials and Methods

### A. Study Design

**FIGURE 1.**
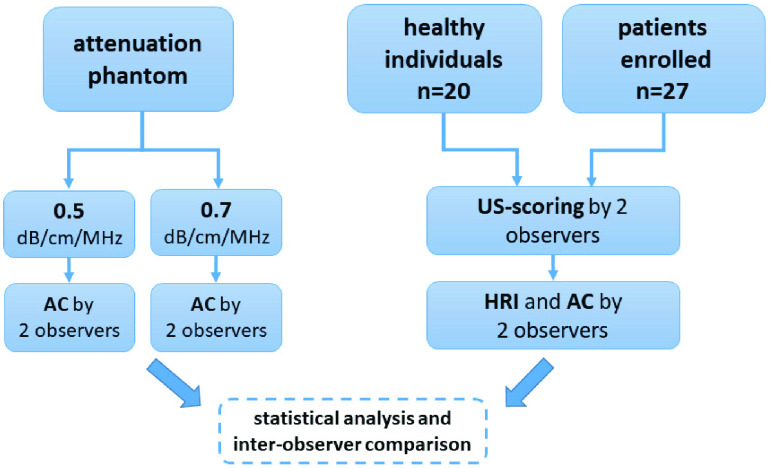
Study flowchart. AC: attenuation coefficient. HRI: hepatorenal index.

The study design is outlined in the flowchart below (Fig. 1). Our institutional review board approved the study (application-number 4259, amendment 359_18 B).

### B. Phantom Measurements

We used the “Sono 406 LE Dual Attenuation Phantom” by Gammex® (Middleton, Wisconsin, USA) as a reference standard for ultrasound attenuation measurements. It contains a tissue mimicking gel, which is ultrasonically similar to human tissue. The phantom features ACs of 0.5 and 0.7 dB/cm/MHz respectively in a side-by-side configuration, thus performing the function normally done by two separate phantoms. Both observers measured the ACs five times on both sides of the phantom at depths of 40 mm and 80 mm independently of each other.

### C. Study Participants

Patients with abnormal liver blood tests, who were referred to our center for a liver parenchyma biopsy between October of 2018 and November of 2019 were included in this study. They had to be more than 18 years of age and give informed consent before the ultrasound examination. Exclusion criteria were the known presence of liver cirrhosis based on patient history or ultrasound findings and the potentially harmful use of alcohol, defined as an intake of more than 20 g for women and 40 g for men [14]. The following patient characteristics were assessed: age, sex, height, weight, waist-to-hip-ratio, daily intake of alcohol and the presence of arterial hypertension, diabetes mellitus or metabolic syndrome. The NAFLD-fibrosis-score [15] was calculated using laboratory data. We also included 20 healthy volunteers as a control group, who did not have any medical history of liver disease or metabolic syndrome.

### D. Ultrasound Analysis

Ultrasound was performed either directly before the liver biopsy or on the following day. Patients were examined with the Aplio i900®-ultrasound machine (Canon Medical Systems, Otawara, Japan) in a supine position, after they had fasted for at least four hours. Two specialists in internal medicine, who had at least three years of experience in abdominal ultrasound, examined the patients independently of each other. First, a complete ultrasound scan of the liver in the longitudinal and horizontal plane was conducted, including the measurement of the liver size by adding the ventrodorsal and craniocaudal diameter in longitudinal view in the right midclavicular line. The observers were blinded to the outcome of the liver biopsy and the results of the ultrasound examination the second observer. The order, in which the ultrasound examinations were performed, was randomized.

*Conventional Ultrasound Scoring:*Both observers assessed the following B-mode image criteria: the brightness of liver in relation to kidney parenchyma, the blurring of liver vessel walls and the attenuation of the ultrasound signal in the posterior segments of the liver. According to these criteria, the grade of fatty infiltration was estimated on and ordinal scale from zero to three according to scoring systems used by other authors [3]. Hence, a mere elevation of liver parenchyma brightness compared to the kidney was considered to represent mild (S1) steatosis. Additional blurring of vessel walls and posterior shadowing of liver parenchyma with the diaphragm still visible was defined as moderate (S2) steatosis. If the diaphragm was not visible due to posterior shadowing on top of the other criteria, the observers assumed severe steatosis (S3).

### E. Hepatorenal Index (HRI)

**FIGURE 2.**
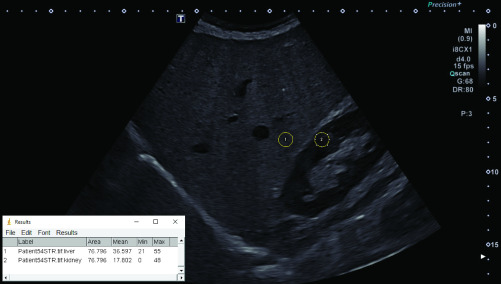
Measurement of the HRI in a 79 year old patient with S1-steatosis (fatty infiltration 30%, HRI 2.06).

For the calculation of the HRI, a B-mode image visualizing liver and kidney parenchyma through an intercostal sonic window was saved using the DICOM (Digital Imaging and Communications in Medicine)-format. To acquire this image, we always used the standard preset for abdominal ultrasound (convex probe with 4 MHz) with the gain control optimized by the machine. We then exported the image as a “Tagged Image File Format” (TIFF)-file with a resolution of 1280 x 960 pixels and analyzed it offline with the “ImageJ® ”-software developed by the National Institutes of Health (Bethesda, USA). This technique has been used in other scientific studies [16]. For the analysis, a round-shaped region of interest (ROI) with a diameter of 10 mm was placed in the kidney and liver parenchyma at the exact same image depth. Large vessels, bile ducts, focal liver lesions and kidney calyxes were avoided. Mean grayscale intensity in the ROI was measured with the software and the HRI calculated by dividing the mean intensity of liver by the mean intensity of kidney parenchyma (Fig. 2).

### F. Attenuation Imaging

**FIGURE 3.**
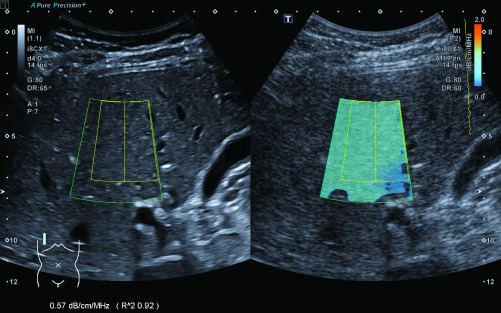
Measurement of the AC in a 28 year-old healthy individual without fatty infiltration of the liver.

**FIGURE 4.**
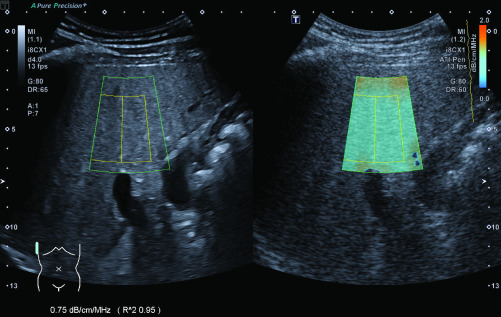
Measurement of the AC in a 34 year-old patient with S3-steatosis (fatty infiltration >70%).

We conducted the AC-measurements according to the manufacturer’s instructions [17]: The ultrasound probe was placed perpendicular to the patient’s skin in an intercostal space above the right liver lobe. The box for the color mapping of the AC, which is shaped like the outer segments of a circle with a size of 50 x 40 mm, was then placed in the liver parenchyma avoiding any large vessels, bile ducts and artifacts from the capsule, which are indicated by orange in the color map. The depth for the measurements was chosen to be as shallow as possible, while still avoiding reverberation artifacts, which is approximately 15–20 mm below the capsule. Once the box had been placed correctly, the measurement was performed by freezing the screen and selecting a ROI with a size of 35 x 30 mm within the color-mapped area. The coefficient of determination (R^2^) for the measurement, which is shown on screen by the ultrasound machine, had to be greater than 0.9 for each measurement; otherwise, the measurement was disregarded. Example images for the measurement of ACs are shown in Fig. 3 (normal liver parenchyma) and Fig. 4 (hepatic steatosis). According to the manufacturer’s instructions and machine settings, the median AC was calculated out of five separate box placements and measurements. This median was used for further statistical analysis. In addition, we measured the distance between the skin and the liver capsule as well as the distance between the capsule and the box for AC-measurements.

### G. Liver Biopsy and Histological Analysis

Liver biopsies were performed under continuous ultrasound visualization using a 16- or 18-gauge needle yielding a sample of 22 mm in length. All specimen were taken from the right liver lobe in segments VI-VIII. Since there was a delay between the AC-measurements and the biopsy, we could not ensure that both had been performed at the exact same location. Liver steatosis was categorized according to guidelines [18]: absence of steatosis S0 (< 5% fatty infiltration), mild steatosis S1 (5–32%), moderate steatosis S2 (33–66%), and severe steatosis S3 (>66%). Fibrosis was determined using the Ishak fibrosis score with seven stages (F0-F6) [19]. Diagnosis of NASH was reached using the SAF-Score with a semi quantitative evaluation of steatosis, hepatocellular ballooning and lobular inflammation [20].

### H. Statistical Analysis

Values shown are the mean ± standard deviation and range in parentheses, if not indicated otherwise. For all tests, a p-value < 0.05 was considered to be statistically significant. For AC and HRI-measurements, the mean of the two observers was used for further statistical analysis. Phantom measurements: For the tests performed on the phantom, we compared measurements taken at 40 and 80 mm depth using the non-parametric Mann-Whitney U test. Furthermore, the measurements taken by each observer were compared with the reference AC-value of the phantom as the theoretical mean in a one sample t-test. To quantify differences between the observers we calculated the intraclass correlation coefficient (ICC). Study population: We compared the differences in the characteristics of patients and healthy individuals with the Mann-Whitney U test. For categorical data we used Fisher’s exact test. Correlation between HRI and AC-values and different patient characteristics was analyzed using Spearman’s rank-order correlation. For statistically significant correlations between patient characteristics and AC/HRI-values, we performed a multivariate linear regression analysis.

All statistical analyses were performed using Version 21 of SPSS® Statistics by IBM (Armonk, New York, USA) or Version 8 of Prism® by GraphPad Software (San Diego, California, USA).

## III. Results

### A. Measurements in the Attenuation Phantom

The mean ACs measured by the two observers at depths of 40 and 80 mm on both sides of the attenuation phantom with reference ACs of 0.5 and 0.7 dB/cm/MHz are shown in table 1. At 80 mm depth, both observers measured ACs significantly lower than the reference of 0.7 dB/cm/MHz. The ICC between the measurements taken by the two observers was 0.96 at a depth of 40 mm and 0.87 at a depth of 80 mm.

**TABLE 1:** Phantom Measurements

Depth	Phantom AC	Observer A	$\boldsymbol {p}$	Observer B	$\boldsymbol {p}$	ICC
40 mm	0.5 dB/cm/MHz	0.51 ± 0.02	0.43	0.51 ± 0.02	0.43	0.96
	0.7 dB/cm/MHz	0.69 ± 0.04	0.45	0.63 ± 0.04	0.02	
80 mm	0.5 dB/cm/MHz	0.50 ± 0.01	0.37	0.47 ± 0.02	0.04	0.87
	0.7 dB/cm/MHz	0.63 ± 0.03	0.01	0.60 ± 0.04	0.007	

### B. Measurements in Healthy Individuals

Characteristics of the healthy volunteers can be found in supplementary table 1. The mean AC measured in healthy individuals was 0.6 ± 0.04 dB/cm/MHz (95%-CI 0.48-0.67 dB/cm/MHz). The mean HRI in healthy individuals was 1.13 ± 0.14 (95%-CI 0.91-1.42). There was no significant correlation with age, gender, weight, height, body mass index (BMI), skin-capsule distance and liver size for both techniques in the multivariate testing.

### C. Measurements in Patients

#### 1) Patient Characteristics

Out of 27 patients, five suffered from diabetes, nine from arterial hypertension and three fulfilled the diagnostic criteria of metabolic syndrome. The most common diagnoses found in the histological workup of the biopsies were autoimmune hepatitis and NASH (n = 8 each). Further underlying diseases included drug-induced liver injury (n = 3), NAFLD (n = 2), non-specific hepatitis (n = 2), and primary biliary or sclerosing cholangitis (n = 1 each). In two patients no pathology of the liver parenchyma could be found. Results are summarized in table 2 and table 3.

**TABLE 2:** Patient Characteristics

Patient characteristics (n=27)	
Age (years) mean, range	50 ± 17 (18–79)
Gender (Male/female)	14/13
Height (cm)	173 ± 9 (155–195)
Weight (kg)	78 ± 23 (48–153)
BMI (kg/m^2^)	26 ± 6 (18–42)
BMI 25 - < 30 kg/m^2^	8
BMI ≥30 kg/m^2^	6
Waist-to-hip ratio	0.98 ± 0.08 (0.86–1.17)
Skin-capsule-distance (mm)	18 ± 5 (10–36)
Capsule-ROI-distance (mm)	15 ± 3 (9–21)
Skin-ROI-distance (mm)	33 ± 7 (22–51)
Liver size in MCL (cm)	26 ± 3 (21–34)
S0/S1/S2/S3 (histology)	13/4/4/6
NASH/NAFLD	8/2

**TABLE 3:** Histological Fibrosis Score (Ishak)

Ishak fibrosis score	
F0	8
F1	12
F2	3
F3	3
F4	1
F5	0
F6	0

#### 2) Conventional Ultrasound Scoring

With conventional US-scoring we predicted the grade of steatosis correctly in 15 out of 27 patients (56%). In the detection of steatosis of any grade, only one out of thirteen patients (8%) without fatty infiltration (S0) was misdiagnosed as having moderate steatosis (S2). Four out of ten patients with hepatic steatosis (S1-3) were classified incorrectly as not having fatty infiltration (S0). This results in a sensitivity of 71% and a specificity of 92% for detecting hepatic steatosis of any grade. Results are shown in table 4.

**TABLE 4:** Conventional Semi Quantitative US-Scoring

		US-scoring			
		S0	S1	S2	S3
Histrology	S0	12	0	1	0
	S1	3	1	0	0
	S2	1	1	2	0
	S3	0	3	3	0

#### 3) Detection of Different Degrees of Steatosis With Attenuation Imaging

**FIGURE 5.**
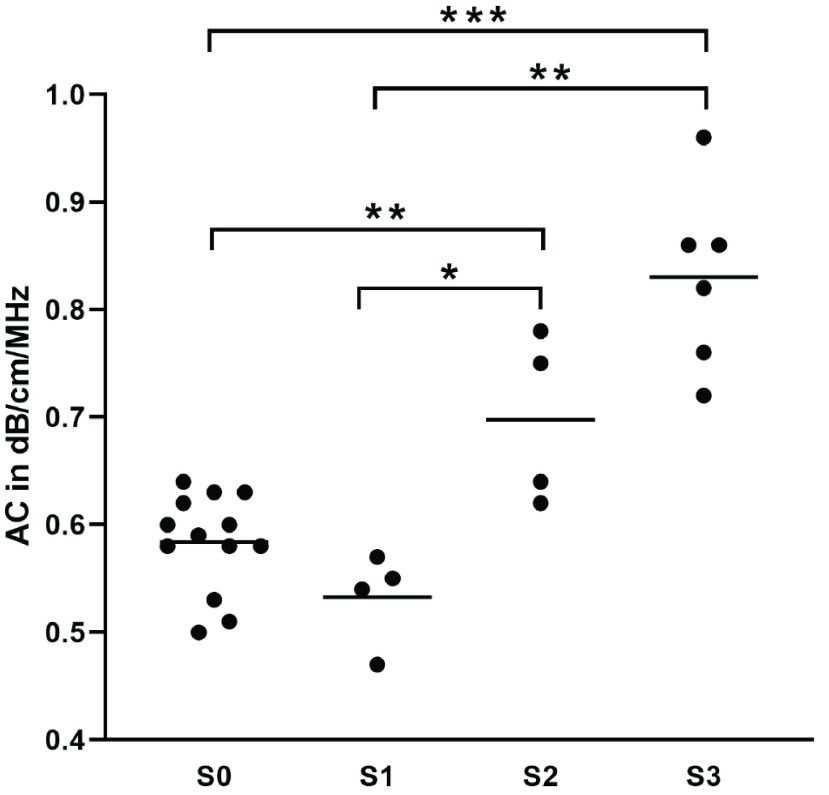
Median ACs in different grades of hepatic steatosis. * p ≤0.05, ** p ≤0.01, *** p ≤0.001.

**FIGURE 6.**
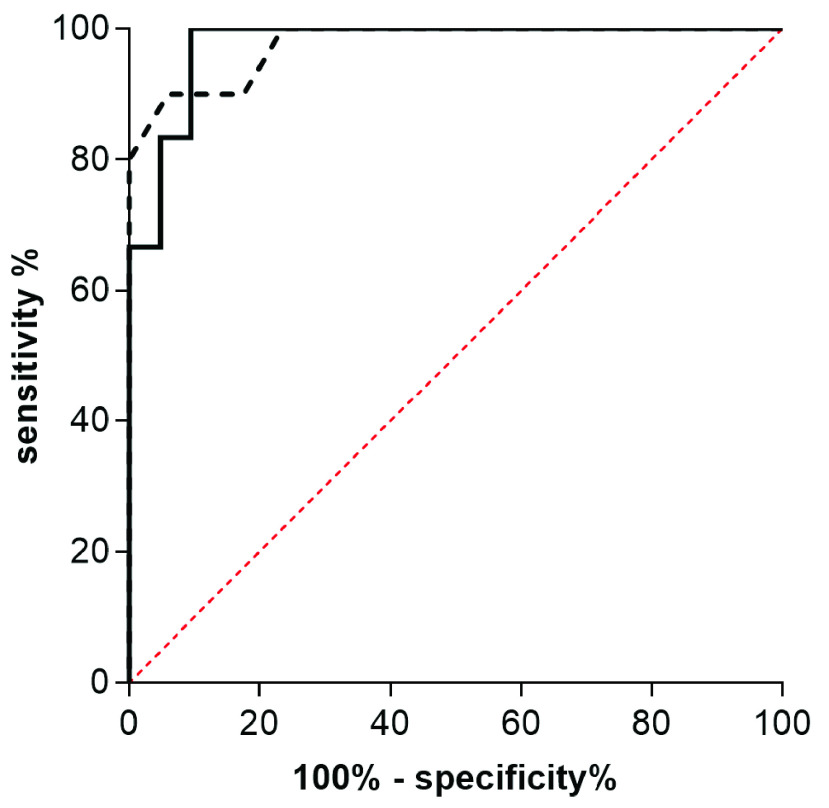
AUROC of ACs. The dotted line represents the detection of hepatic steatosis S≥2, the solid line represents detection of S3.

AC-measurements with an R^2^-value >0.9 could be achieved in all patients. The mean AC in patients without fatty infiltration of liver parenchyma (S0) was 0.58 ± 0.05 dB/cm/MHz. In patients with S1, S2 and S3-steatosis, the mean ACs were 0.53 ± 0.04 dB/cm/MHz, 0.69 ± 0.08 dB/cm/MHz and 0.83 ± 0.08 dB/cm/MHz, respectively. The difference between patients with severe and no hepatic steatosis (S3/S0) and the difference between patients with severe and mild steatosis (S3/S1) were statistically significant (p < 0.0001, p + 0.009). This is also true for the difference between S2 and S0 as well as S2 and S1 (p < 0.009, p = 0.03). Results are shown in Fig. 5. In the Receiver Operating Characteristic (ROC)-analysis, the area under the curve (AUC) was 0.98 for the detection of both ≥ S2 and S3. The cut-off-values according to Youden’s index were 0.64 dB/cm/MHz for the detection of steatosis ≥S2 and 0.68 dB/cm/MHz for S3. With these cut-off values the sensitivity for detecting a steatosis ≥S2 was 90% with a specificity of 94%. For detecting S3- steatosis the sensitivity was 100% with a specificity of 90%. ROC-curves for the detection of moderate and severe steatosis are shown in Fig. 6.

#### 4) Detection of Different Degrees of Liver Steatosis With the HRI

**FIGURE 7.**
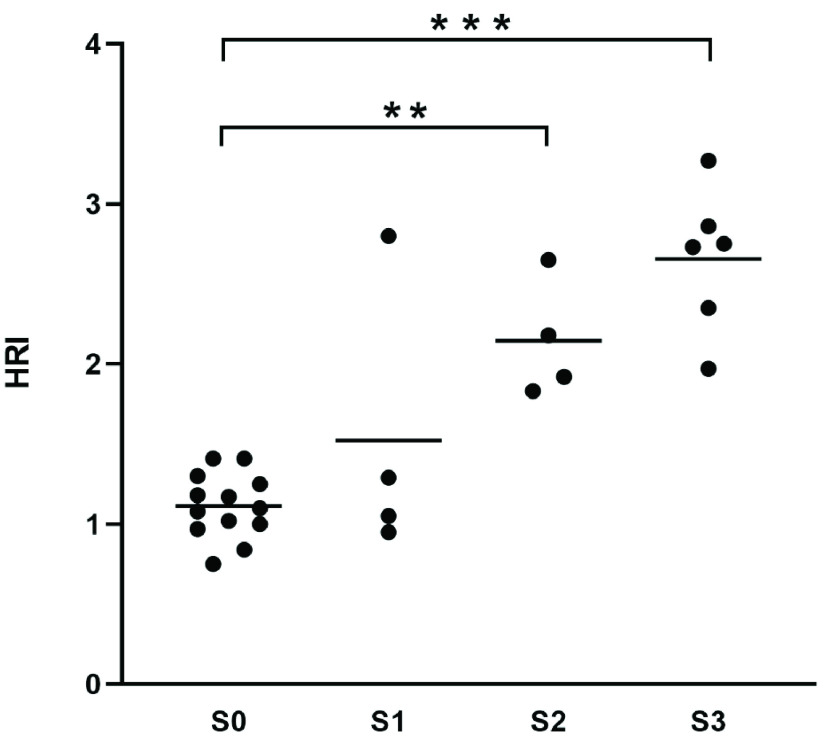
Median HRI-values in different grades of hepatic steatosis. ** p ≤0.01, *** p ≤0.001.

**FIGURE 8.**
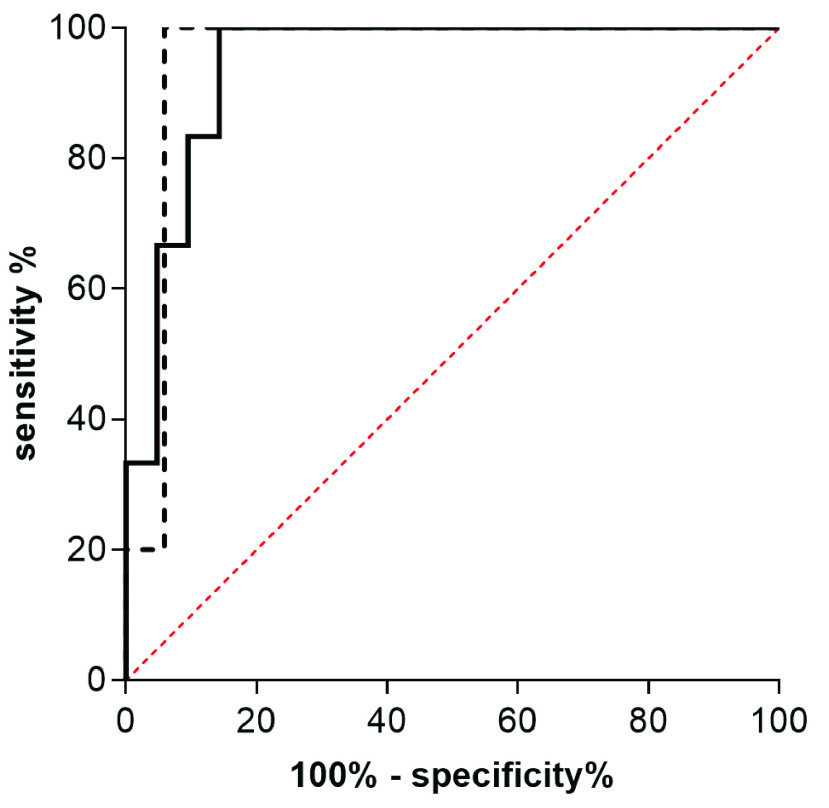
AUROC of HRI-values. The dotted line represents the detection of hepatic steatosis S≥2, the solid line represents detection of S3.

The mean HRI for the different grades of steatosis was 1.11 ± 0.2 for S0, 1.52 ± 0.86 for S1, 2.15 ± 0.37 for S2 and 2.66 ± 0.45 for S3. Only the differences between patients with S2 and S3-steatosis in comparison to patients without fatty infiltration (S0) were statistically significant (p < 0.0001 and p = 0.0008). Results are shown in Fig. 7. In the ROC-analysis the AUC-value was 0.95 for the detection of a steatosis ≥ S2 and 0.94 for the detection of S3-steatosis. The optimal cut-off value calculated with Youden’s index was 1.62 for the detection of S≥2 and 1.95 for S3. Sensitivity and specificity with these cut-off-values were 100% and 94% for detecting moderate (S2) and 100%/86% for detecting severe steatosis (S3). ROC-curves for the detection of moderate and severe steatosis are shown in Fig. 8.

#### 5) Correlation With Patient Characteristics

The ACs showed a significant correlation with the degree of steatosis ($\rho = 0.65$; p < 0.001). In addition, there was a negative correlation with height ($\rho =-0.45$; p = 0.03) in the univariate analysis, but not with any of the other patient characteristics we assessed, including skin-capsule and capsule-ROI-distance. In the multivariate analysis, height turned out to be a non-significant factor. Results can be found in supplementary table 2. For the HRI, we observed a significant correlation with the degree of steatosis ($\rho =0.73$, p < 0.001), skin-capsule-distance ($\rho =0.43$; p = 0.03) and liver size ($\rho = 0.47$; p = 0.02) in the univariate analysis. Skin-capsule-distance and liver size turned out to be not significant in the multivariate testing. For detailed results see supplementary table 3.

### D. Inter-Observer Agreement

#### 1) Inter-Observer Agreement of Attenuation Imaging

In healthy individuals, the mean difference of the AC-medians measured by the two observers was 0.06 dB/cm/ MHz (95%-CI 0.04-0.08 dB/cm/MHz), which is 10% (95%-CI 6.6-13.5%) of the mean. In patients, the mean difference between the median ACs measured by the two observers was 0.07 dB/cm/MHz (95%-CI 0.03-0.09 dB/cm/MHz) or 9.8% (95%-CI 6.7-13%) of the mean. The ACs measured by the two observers showed a highly significant Spearman’s rank correlation with a coefficient of 0.74 (95%-CI 0.48 to 0.88, p < 0.001) and a very good ICC of 0.92.

#### 2) Inter-Observer Agreement of the HRI

In healthy individuals, the mean difference of calculated HRI-values was 0.17 (95%-CI 0.11-0.22), which is 14% of the mean (95%-CI 10-18.7%). In patients, the mean difference was 0.23 (95%-CI 0.14-0.33) or 15% of the mean (95%-CI 10.6-20.1%). The Spearman’s rank correlation coefficient was 0.88 (95%-CI 0.74 to 0.95, p < 0.001) and the ICC was excellent (0.94).

## IV. Discussion

Noninvasive imaging techniques for the quantification of liver fat have been the subject of scientific research for decades. Although MRI (magnetic resonance imaging)-based techniques have shown good diagnostic performance, their use in daily clinical practice is limited due to their time-consuming nature and high cost. Ultrasound is the method of choice for detecting hepatic steatosis as recommended in guidelines [21]. Quantification of steatosis by ultrasound B-mode image criteria has been shown to correlate with histological results, but with low diagnostic accuracy and inter-observer agreement [5]. Its sensitivity and specificity are high for the detection of moderate and severe steatosis (85% and 94%), but significantly lower for mild steatosis (sensitivity approximately 60%) [22]. In our study, we also had difficulties quantifying liver steatosis by B-mode image criteria correctly, especially in patients with moderate and severe steatosis (S2/3). The need for an ultrasound technique, which can detect and quantify fatty infiltration of the liver objectively and with high accuracy is therefore still unmet. The HRI is a more objective technique than conventional US-scoring and has shown promising results in the detection and quantification of fatty liver when compared to MR-PDFF (proton density fat fraction) as the gold standard. Sensitivity and specificity for detecting different degrees of steatosis have been reported to be >90% and >84%, respectively [23], [6]. Histology-based studies, on the other hand, have shown lower diagnostic accuracy of the HRI, some of them with a lack of statistical significance for differentiating single grades of fatty liver. Additionally, it is not clear which cut-off value should be used to detect hepatic steatosis, proposed values range from 1.24 to 2.2 and the HRI has therefore not found its way into daily clinical practice [16], [24], [25]. Attenuation imaging is a novel technique, which has recently been integrated into an ultrasound machine to objectively detect and quantify hepatic steatosis. Studies comparing its diagnostic performance in patients with histology as the gold standard are limited, but have shown good correlation with fatty infiltration of the liver parenchyma and moderate to good AUROC-values for the quantification of different grades of hepatic steatosis [10], [11]. In our current study, we focused on the inter-observer variability of attenuation imaging and compared its diagnostic capabilities with the HRI.

Our measurements in the attenuation phantom showed excellent inter-observer reliability with an ICC of 0.96 for measurements at 40 mm depth, where the technique is most commonly used. At 80 mm depth, the ICC decreased but was still very good (0.87). The measurements of both observers also differed significantly from the reference AC of 0.7 dB/cm/MHz at a depth of 80 mm, which might suggest that the diagnostic accuracy decreases with a higher distance of the probe to the ROI. Indeed, the diagnostic performance has been shown to decrease in obese patients in previous studies [11]. In our study, we did not find statistically significant correlations of the ACs with patient weight, BMI or skin-ROI-distance. The range of the skin-ROI-distance in our patient cohort reached from 22 to 47 mm, so we did not measure ACs at a depth where the phantom measurements had shown a decrease in accuracy, which might explain these seemingly contradictory findings.

Regarding the diagnostic performance of attenuation imaging in comparison with the HRI, both techniques correlated significantly with the degree of steatosis in our study ($\rho =0.65$ for ACs and $\rho =0.77$ for the HRI), but not with any other patient characteristics, especially not with the grade of fibrosis. This is unexpected, since studies in the past had already shown increased attenuation of the ultrasound beam in fibrotic liver parenchyma [26]. A recent study, which used the same ultrasound machine, also found a significant correlation between AC-values and fibrosis, although they used MR-PDFF instead of liver biopsy as the reference standard [9]. In our cohort, almost all of the patients did not have significant fibrosis (23 out of 27 patients with F0-F2), which could explain this difference. On the other hand, two studies with a higher ratio of patients with significant fibrosis did not see a correlation between fibrosis and AC-values [10], [13]. Future trials will tell, whether the effects of fibrosis on attenuation imaging are clinically relevant.

Both attenuation imaging and the HRI were unable to discriminate between S0- and S1-steatosis in our study, whilst the AUROC-values for detecting moderate (S2) and severe (S3) steatosis were excellent for both methods (0.98 for ≥S2 and S3 with ACs; 0.95 for ≥S2 and 0.94 for S3 with HRI). These findings confirm results of previous studies by other authors, which had also shown good performance in the detection of moderate and severe, but not mild steatosis with attenuation imaging. In contrast, these studies had been able to detect a statistically significant difference between ACs of patients with S0 and S1-steatosis [10]–[13]. This discrepancy is most likely due to the low number of patients with S1-steatosis (n = 4) in our study population.

Despite the small sample size of our cohort, the cut-off AC-values we calculated for different grades of steatosis (0.64 dB/cm/MHz for ≥S2 and 0.68 dB/cm/MHz for S3) are very similar to the numbers published by Tada et al (0.67 dB/cm/MHz for ≥S2 and 0.68 dB/cm/MHz for S3) [11]. The values calculated by Bae *et al.* (0.700 dB/cm/MHz for ≥S2 and 0.745 dB/cm/MHz for S3) [10] and Burgio *et al.* are slightly higher (0.72 dB/cm/MHz for ≥S2) [13]. In all of these studies, there was a significant area of overlap between the different groups of steatosis.

Overall, we could confirm that attenuation imaging is a promising tool for the detection and quantification of moderate and severe liver steatosis. Measurements of ACs were not influenced by other important patient characteristics in our study. The technique also showed very good inter-observer reliability. Although we did not find a statistically significant advantage of attenuation imaging in comparison to the HRI, some advantages remain from a clinical viewpoint: when measuring ACs, only liver parenchyma has to be visualized in the sonic window, which is easier to perform and possible changes of echogenicity of the kidney parenchyma do not influence the results of the measurement. This is especially important in patients with NAFLD, who show an increased risk of developing chronic kidney disease [27]. The question of whether the accuracy of AC-measurements decreases with increasing depth of the ROI will have to be addressed in future studies which include more patients, which are obese.

Limitations of our study are the low number of patients, especially patients with mild steatosis (S1) and the heterogeneity of the study population in regard to the underlying liver disease. A more uniform cohort consisting only of patients with NAFLD would be desirable. Also, it cannot be ruled out, that there is a significant sampling error in some of the histological findings, since we could not ensure that all liver biopsies were taken from the exact same area where the AC-measurements had been conducted.

## V. Conclusion

Attenuation imaging is a new and exciting method, which has still to be tested in larger trials. We think it could especially be useful for follow-up studies of patients with moderate or severe steatosis undergoing lifestyle changes or pharmacological treatment of NAFLD.

## VI. Conflicts of Interest

D.J. and D.S. received speaker honorarium by Canon Medical Systems.

## VII. Source of Funding

This study has not received any specific funding to declare.
